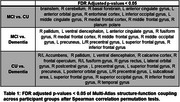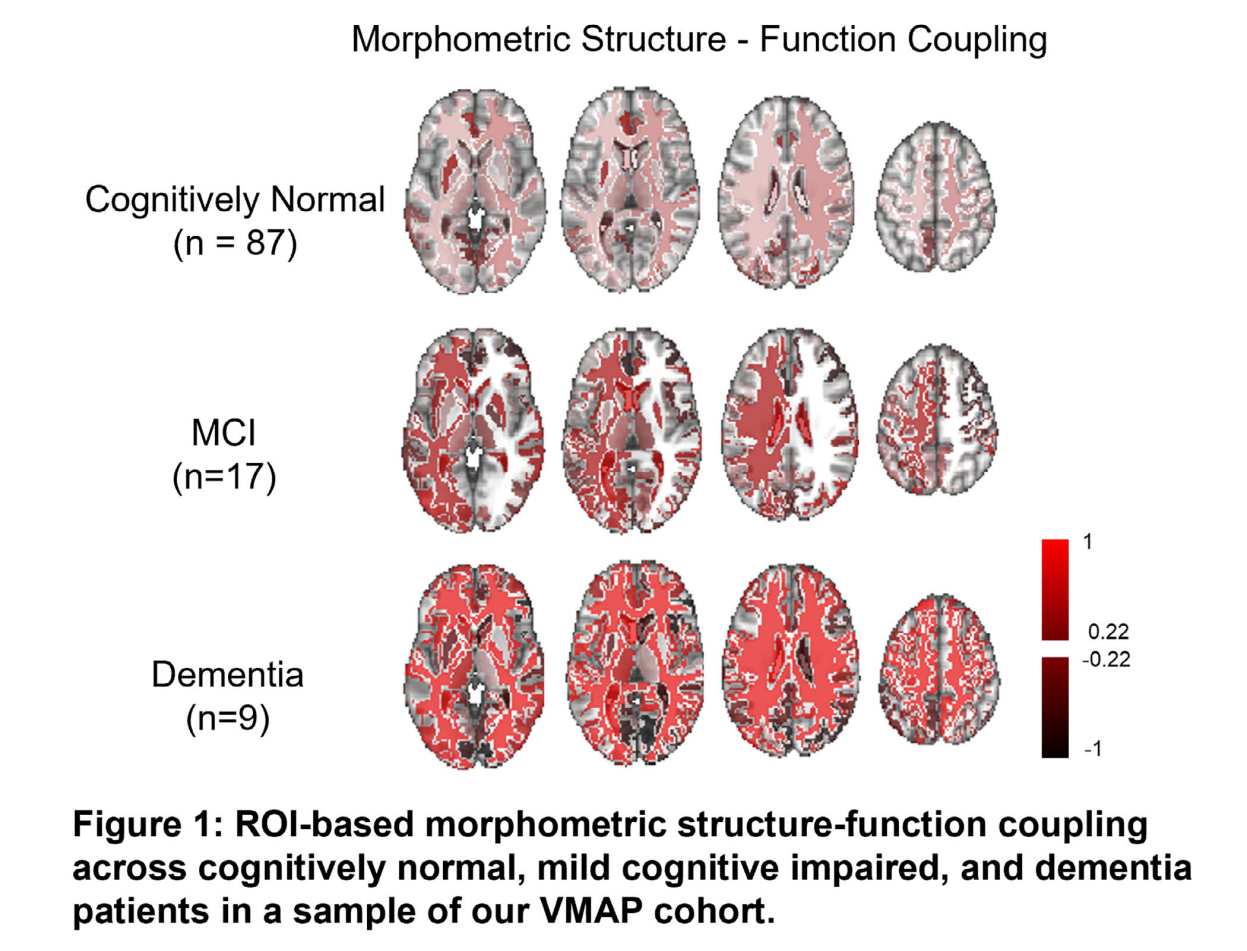# Morphometric Volume‐Function Coupling Across the Clinical Trajectory of Dementia

**DOI:** 10.1002/alz70856_099383

**Published:** 2025-12-24

**Authors:** Sarah E Goodale, Panpan Zhang, Yukti Vyas, Kimberly R. Pechman, Niranjana Shashikumar, Bennett A. Landman, Timothy J. Hohman, Angela L. Jefferson

**Affiliations:** ^1^ Vanderbilt Memory & Alzheimer's Center, Vanderbilt University Medical Center, Nashville, TN, USA; ^2^ Department of Biostatistics, Vanderbilt University Medical Center, Nashville, TN, USA; ^3^ Vanderbilt Memory and Alzheimer's Center, Vanderbilt University School of Medicine, Nashville, TN, USA; ^4^ Department of Computer Science, Vanderbilt University, Nashville, TN, USA; ^5^ Department of Electrical and Computer Engineering, Vanderbilt University, Nashville, TN, USA; ^6^ Department of Neurology, Vanderbilt University Medical Center, Nashville, TN, USA; ^7^ Department of Neurology, Vanderbilt Memory & Alzheimer's Center, Vanderbilt University Medical Center, Nashville, TN, USA; ^8^ Department of Medicine, Vanderbilt University Medical Center, Nashville, TN, USA

## Abstract

**Background:**

Brain structure and function alterations in aging are associated with cognitive decline and Alzheimer's disease and related dementias (ADRD). Characterizing the spatial distribution through which total brain volume and functional connectivity interact across the cognitive spectrum may provide additional insights into ADRD progression.

**Methods:**

A subset of Vanderbilt Memory and Aging Project participants (*n* = 113, 75.7±6.2years, 34% female, *n* = 87 cognitively unimpaired (CU), *n* = 17 mild cognitively impaired (MCI), and *n* = 9 dementia) underwent functional magnetic resonance imaging (MRI) and T1‐weighted MRI at 3T. Morphometric volumes across the grey and white matter were calculated using Multi‐Atlas (MA) segmentation, and functional connectivity was calculated using Pearson correlation and a fisher‐Z transform in the same MA regions. Volumetric and functional data underwent permutation testing (*n* = 10000) via Spearman correlation to create a morphometric structure‐function coupling metric covarying for age, sex, ethnicity/race, education, and intracranial volume. Region‐based multiple comparisons were corrected for using false discovery rate (FDR).

**Results:**

Structure‐function coupling showed significant differences across the brain in dementia compared to both CU and MCI as well as between MCI and CU participants. The spatial distribution across all groups and a list of significant regions after FDR adjusted *p*‐values<0.05 are illustrated in Figure 1 and summarized in Table 1 respectively. Focusing on adjusted *p*‐values<0.01, MCI and CU participants showed differences in the left anterior cingulate, left inferior occipital gyri, and the right entorhinal cortex. Dementia and CU participants revealed differences in the left superior frontal and lateral orbital gyri and right precentral gyrus. Finally, MCI and dementia participants had differences between the right pallidum, medial frontal cortex, and precentral gyrus, as well as the left anterior cingulate gyrus.

**Conclusions:**

Morphometric structure‐function coupling has widespread increases with worse cognitive status as seen by our significant results across CU, MCI, and dementia participants. These stronger, correlative couplings in MCI and dementia could be due to regional atrophy and disrupted functional connectivity, which causes the relationship between the two variables to be more pronounced as compensatory capacity within the brain is diminished. Future work will incorporate an increased sample size for sufficient power and will evaluate potential cardiovascular influences on the structure‐function coupling.